# Age-related changes to vestibular heave and pitch perception and associations with postural control

**DOI:** 10.1038/s41598-022-09807-4

**Published:** 2022-04-19

**Authors:** Grace A. Gabriel, Laurence R. Harris, Joshua J. Gnanasegaram, Sharon L. Cushing, Karen A. Gordon, Bruce C. Haycock, Jennifer L. Campos

**Affiliations:** 1 KITE-Toronto Rehabilitation Institute, University Health Network, Toronto, ON Canada; 2 Department of Psychology, University of Toronto, 500 University Avenue, Toronto, ON M5G 2A2 Canada; 3 Department of Psychology and Centre for Vision Research, York University, Toronto, ON Canada; 4 Department of Otolaryngology-Head and Neck Surgery, Hospital for Sick Children, Toronto, ON Canada; 5 Department of Otolaryngology-Head and Neck Surgery, University of Toronto, Toronto, ON Canada; 6 Archie’s Cochlear Implant Laboratory, Hospital for Sick Children, Toronto, ON Canada; 7 University of Toronto Institute for Aerospace Studies, Toronto, ON Canada

**Keywords:** Psychology, Human behaviour, Sensory processing

## Abstract

Falls are a common cause of injury in older adults (OAs), and age-related declines across the sensory systems are associated with increased falls risk. The vestibular system is particularly important for maintaining balance and supporting safe mobility, and aging has been associated with declines in vestibular end-organ functioning. However, few studies have examined potential age-related differences in vestibular *perceptual* sensitivities or their association with postural stability. Here we used an adaptive-staircase procedure to measure detection and discrimination thresholds in 19 healthy OAs and 18 healthy younger adults (YAs), by presenting participants with passive heave (linear up-and-down translations) and pitch (forward–backward tilt rotations) movements on a motion-platform in the dark. We also examined participants’ postural stability under various standing-balance conditions. Associations among these postural measures and vestibular perceptual thresholds were further examined. Ultimately, OAs showed larger heave and pitch detection thresholds compared to YAs, and larger perceptual thresholds were associated with greater postural sway, but only in OAs. Overall, these results suggest that vestibular perceptual sensitivity declines with older age and that such declines are associated with poorer postural stability. Future studies could consider the potential applicability of these results in the development of screening tools for falls prevention in OAs.

## Introduction

Falls are the most common cause of fatal and non-fatal injuries in adults over the age of 65 years^1,2^ and age-related declines across sensory systems (e.g., vision, hearing, proprioception, vestibular) contribute to an increased risk of falls. The vestibular system is particularly important for maintaining balance and supporting safe mobility^3,4^. It is well established that vestibular functioning changes with age as evidenced by physiological changes and changes to peripheral vestibular end-organ measures^5–7^. There are also well-known age-related behavioral changes, such as declines in postural control, that occur with older age^8–14^. However, much less is known about age-related changes to vestibular *perception* (but see ^15^ for a review) and how vestibular perceptual abilities are associated with postural stability in older adults^16–21^. In this study, we examine age-related differences in vestibular detection and discrimination thresholds during passive heave and pitch movements in the dark, and explore how these percepts are associated with posturography measures (i.e., center of pressure path length, velocity, root-mean-squared velocity).

### Age-related changes in vestibular end-organ structure and functioning

The vestibular system comprises five distinct organs: three semicircular canals that detect rotational motion in all three axes (yaw, pitch, and roll), as well as two otolith organs that detect linear acceleration and gravity in the vertical or heave axis (saccule), and in the horizontal, or surge and sway axes (utricular, and some saccular)^22^. These vestibular end organs send sensory information to the brain through the vestibulocochlear nerve, which informs the perception of self-motion^23^ for a review).

Age-related changes to the vestibular system have been evaluated by examining the vestibular end organs histologically and microscopically^24–26^ (but see ^5–7,27^ for reviews). These studies have demonstrated that aging is associated with declines in end organ integrity, including deterioration of the otoconia in the otolith organs, particularly in the utricle^28^ and a loss of Type I^29^ and Type II hair cells^24,29–32^, with the semicircular canals being particularly susceptible to Type I hair cell loss^32,33^. There is also evidence of degeneration of the vestibular ganglion with aging^34,35^, especially the superior vestibular nerve^36^ which relays afferent superior (i.e., pitch) and lateral (i.e., yaw) semicircular canal information to the brain.

Within clinical settings, saccular, utricular, and semicircular canal functioning are often assessed using cervical vestibular-evoked myogenic potentials (cVEMP), ocular VEMPs (oVEMP) and video head impulse testing (vHIT), respectively. Reported age-related changes to these measures include decreased cVEMP amplitudes^37,38^ and increased oVEMP latencies^39^, which suggest decline in vestibular functioning. There is also evidence of slightly lower vHIT gains in older adults over 85 years of age compared to younger adults^37,40,41^, although many studies also show that vHIT is resistant to age-related effects^42,43^. Less, however, is understood about how age-related changes to end-organ functioning, as measured by VEMP and vHIT, are associated with age-related changes in self-motion perception or vestibularly-informed behaviors such as balance and postural control^44–47^.

### Age-related changes in postural control and vestibular perception

A wealth of previous research has characterized age-related changes to postural control^48–52^. For instance, older adults demonstrate poorer standing balance (e.g., longer center of pressure, or COP, path lengths and greater sway velocity) during static posturography tasks compared to younger adults, particularly with eyes closed^9^, while standing on compliant or unstable surfaces^11,53^, or during balance perturbations (e.g., ^54^).

During passive movements in the dark, self-motion *perception* is thought to be largely informed through vestibular inputs. Under such conditions younger adults have demonstrated an ability to detect, estimate, and discriminate rotational velocity^17,55^, linear heading direction^20,56–60^, distance travelled^59,61–63^, and target-relative spatial updating^64–69^. There is emerging, but limited evidence suggesting that vestibular perception changes with older age. For instance several studies have shown that, compared to younger adults, older adults demonstrate higher direction-discrimination thresholds for sway, heave, and roll-tilt^15,16,18,19,70,71^ but not yaw^16,70^. These studies have also shown that younger and older adults do not demonstrate differences in their ability to detect movements in yaw, or in their ability discriminate between two passively-applied yaw rotations^17^. Greater age-related differences in direction-discrimination thresholds have been reported for linear movements compared to rotational movements^16^. No studies to our knowledge, however, have examined age-related perceptual changes in the pitch direction, although previous studies have suggested that older adults may have a biased perception of verticality (with “backward disequilibrium syndrome”) and exhibit a backwards-tilted bias in their subjective postural vertical, when compared to healthy control participants^72–74^. As such, examining pitch perception in older adults may be particularly informative given that forward-tilt detection has been described as a potential predictor of falls risk in older adults^75,76^. Likewise, falls and recovery from falls, especially in older adults, may be associated with motion in the z-plane, as elevating and lowering strategies are the most common strategies used to recover from a fall due to tripping^75–78^. Therefore, we also investigated the extent to which there may be age-related differences in the ability to detect, and discriminate between, vertical linear (i.e., heave) motions.

Furthermore, it might be expected that less sensitive vestibular perception may have negative implications for behaviors that are informed by self-motion perception, such as standing balance. Yet, few studies^16,18,19^ have examined the extent to which age-related changes in balance and postural control are associated with measures of vestibular self-motion perception. It has recently been shown that in younger and middle-aged adults, different measures of COP path length are related to vestibular perceptual thresholds for many translational and rotational movements^79^. The results showed that vestibular perceptual thresholds in the lateral plane were positively associated with COP path length. In older adults specifically, some research has indicated that higher roll-tilt thresholds are associated with a greater risk of failing the most difficult condition of a Romberg Balance Test (i.e., quiet stance on a compliant surface with eyes closed^16,18,19^). While failure to successfully complete this type of balance test is an important indicator of poor postural control, the binary nature of “pass” and “fail” tasks could conceal subtler differences or declines in postural stability which may, nonetheless, be important predictors of mobility or falls risk^80–83^. Higher resolution spatial and temporal measures of sway could allow for the examination of finer differences in postural control which may not be severe enough to cause full balance failures. It would also provide the opportunity to relate such differences to vestibular perceptual thresholds in older and younger adults.

To our knowledge, associations between vestibular perceptual thresholds (e.g., direction discrimination, detection, magnitude-discrimination) and more precise spatial or temporal features of postural sway (e.g., COP path length and velocity) have not been examined in healthy older adults. In general, better characterizing vestibular perceptual sensitivities of older adults across a range of motion types and axes, as well as understanding how these perceptual abilities are associated with high resolution measures of posture could help to clarify the extent to which age-related declines in vestibular function (e.g., presbyvestibulopathy^84^) contribute to balance problems and falls risk.

### Current study

In this study, we measured peripheral vestibular end-organ functioning using vHIT and VEMPs in older adults, as well as behavioral balance functioning (posturography during quiet standing) and vestibular perception (two-interval detection and magnitude discrimination thresholds) in younger and older adults. We also examined associations between perceptual thresholds and posturography measures in each age group. Specifically, by passively moving participants using a 6 degrees-of-freedom motion platform we measured movement detection and magnitude discrimination thresholds during heave translation, which stimulates the saccule, and during pitch rotation, which stimulates both anterior and posterior canals, as well as the saccule and utricle. We also used a static posturography task to assess postural stability and performed a series of exploratory correlations between vestibular perceptual thresholds and posturography measures for each age group.

## Methods

### Participants

19 healthy older adults (*M*_age_ = 70.47 years, *SD* = 5.64, range = 65–89 years, 11 females, 8 males) and 18 younger adults (*M*_age_ = 26.00 years, *SD* = 4.27, range = 20–34 years, 13 females, 5 males) completed the study. All participants gave written informed consent. A subset of the older adult participants were included as a control sample in Gabriel et al.^85^. Participants were recruited from the community using posters, social media posts, websites, and through an existing participant database. These individuals were eligible to participate if they did not have a history of stroke, seizure, disabling musculoskeletal disorder, acute psychiatric disorder, dementia, mild cognitive impairment, clinically diagnosed vestibular disorders (e.g., Meniere’s disease), hearing loss, or if they were unable to provide informed consent. All older adults obtained above cut-off scores on the Montreal Cognitive Assessment for mild cognitive impairment (MoCA; i.e., $\ge$ 26 points^86^). All methods in this study were approved by the University Health Network’s Research Ethics Board (Protocol #: 18-6123.0), the University of Toronto Research Ethics Board (Protocol #: 00037394), and The Hospital for Sick Children Research Ethics Board (Protocol #: 1000056920).

### Baseline assessment session tests

Older adult participants first underwent a series of baseline sensory (i.e., hearing and vestibular), cognitive, and balance assessments (Table 1), each described in detail in the following sections.

**Table 1:** Summary of baseline assessments measured in the older adult participants.

Baseline measure	M (*SD*)
**Hearing**	
PTA threshold^a^ (dB HL)	11.29 (5.63)
**Cognition**	
MoCA^b^ (/30 total)	27.39 (1.46)
**Vestibular end-organ**	
vHIT^c^ (total *n*)	10
vHIT (right ear)	0.96 (0.19)
vHIT (left ear)	0.89 (0.15)
cVEMP^d^ (total *n*)	14
cVEMP (present, right ear, *n*)	87%
cVEMP (present, left ear, *n*)	67%
oVEMP^e^ (*n*)	14
oVEMP (present, right ear, *n*)	27%
oVEMP (present, left ear, *n*)	27%
**Balance**	
ABC^f^ (/100%)	94.82 (5.15)
Fell in the last year (*n*)	3
Near fall(s) in last year (*n*)	2
Fear of falling (*n*)	2

^a^PTA = Pure Tone Average; frequencies tested: 500, 1000, 2000, and 4000 Hz, inclusive, with a cut-off threshold above 25 dB HL.

^b^MoCA = Montreal Cognitive Assessment (max score = 30; clinical cut off ≤ 26 pts).

^c^vHIT = Video Head Impulse Test. 9 participants were not able to come back to complete this session. Median gain for 60 ms reported. One participant obtained a median gain below the 0.7 cut off score at 60 ms (they obtained 0.67, in the left ear).

^d^cVEMP = 5 participants were not able to come back to complete this session. Cervical Vestibular Evoked Myogenic Potential.

^e^oVEMP = 5 participants were not able to come back to complete this session. Ocular Vestibular Evoked Myogenic Potential.

^f^ABC = Activities-specific Balance Confidence Scale (max score = 100%).

#### Hearing

Given that declines in vestibular functioning may be associated with age-related hearing loss^85,87–92^ older adult participants were screened for hearing abilities. Audiometric testing was completed as per guidelines established by the International Organization of Standardization (ISO; ^93^). Pure-tone audiometry was used to determine audiometric hearing thresholds using a Grason-Stadler 61 Clinical Audiometer (GSI-61; Grason-Stadler Inc., Eden Prairie, MN) and Telephonics TDH-50P headphones (Telephonics Corporation, Farmindale, NY). Testing was performed in a double-walled sound-attenuating booth (Industrial Acoustics Company, Inc., New York, NY). Frequencies tested were between 250 and 8000 Hz, inclusive. Binaural pure-tone audiometric (PTA) thresholds below 25 dB HL, when averaged across the 500 Hz, 1000 Hz, 2000 Hz, and 4000 Hz frequencies, were considered normal. Three older adult participants could not come into the lab to have their hearing tested, but these older adults had self-reported normal hearing and their vestibular threshold and posturography data did not differ significantly from the rest of the older adults.

#### Cognition

Mild cognitive impairment was screened for using the MoCA. The MoCA is a rapid test designed to screen individuals for mild cognitive impairment. The test assesses general cognitive abilities, by examining several domains of cognitive functioning including attention, executive function, memory, and language and is scored out of a total of 30 points. In this study, level-of-education adjusted scores are reported and all participants obtained a score of 26 or higher (common cut-off for mild cognitive impairment).

#### Vestibular

Vestibular end-organ functioning was assessed using vHIT and VEMPs to measure semicircular canal and otolith organ functioning respectively. vHIT assesses semicircular canal (in this case lateral canals) functioning by measuring (and here, reporting) participants’ vestibulo-ocular reflex (VOR). A gain less than 0.7 suggests impaired semicircular canal functioning^42,94–96^.

VEMPs are a measure of otolith organ functioning that exploit the sound-sensitive fibers contained in the saccule and utricle^97^. cVEMPs were scored as “present”, indicating normal otolith function, if activity presented a P1 peak at 10–25 ms, followed by an N1 trough at 20–40 ms. Absence of this peak was coded as “absent” and indicated potential dysfunction of the otolith organs. oVEMPs were coded as present if the test demonstrated an N1 at 8–20 ms, followed by a P1 between 15 and 30 ms. These latency ranges were based on the possibility that peak latencies may change with older age^7,98,99^.

Details regarding vHIT and VEMP testing procedures can be found in the online Supplementary Information S.1.

#### Balance

Self-reported balance function during day-to-day tasks was measured using the Activities-specific Balance Confidence (ABC) scale^100^. Excellent perceived balance was scored by participants on each item as 100%, and very poor subjective balance as 0%. Posturography tasks were also used to assess standing balance during the experimental session (see details below).

#### Demographics and health history questionnaire

A questionnaire recording the participants’ demographics and medical background was administered. Items included questions regarding education, dizziness, history or presence of vestibular disorders, fear of falls, history of falls, smoking and drinking habits, subjective cognitive decline, heart disease and other vascular or neurological health problems.

### Experimental session

#### Vestibular psychophysics task

##### Stimuli and apparatus

**Figure 1:**
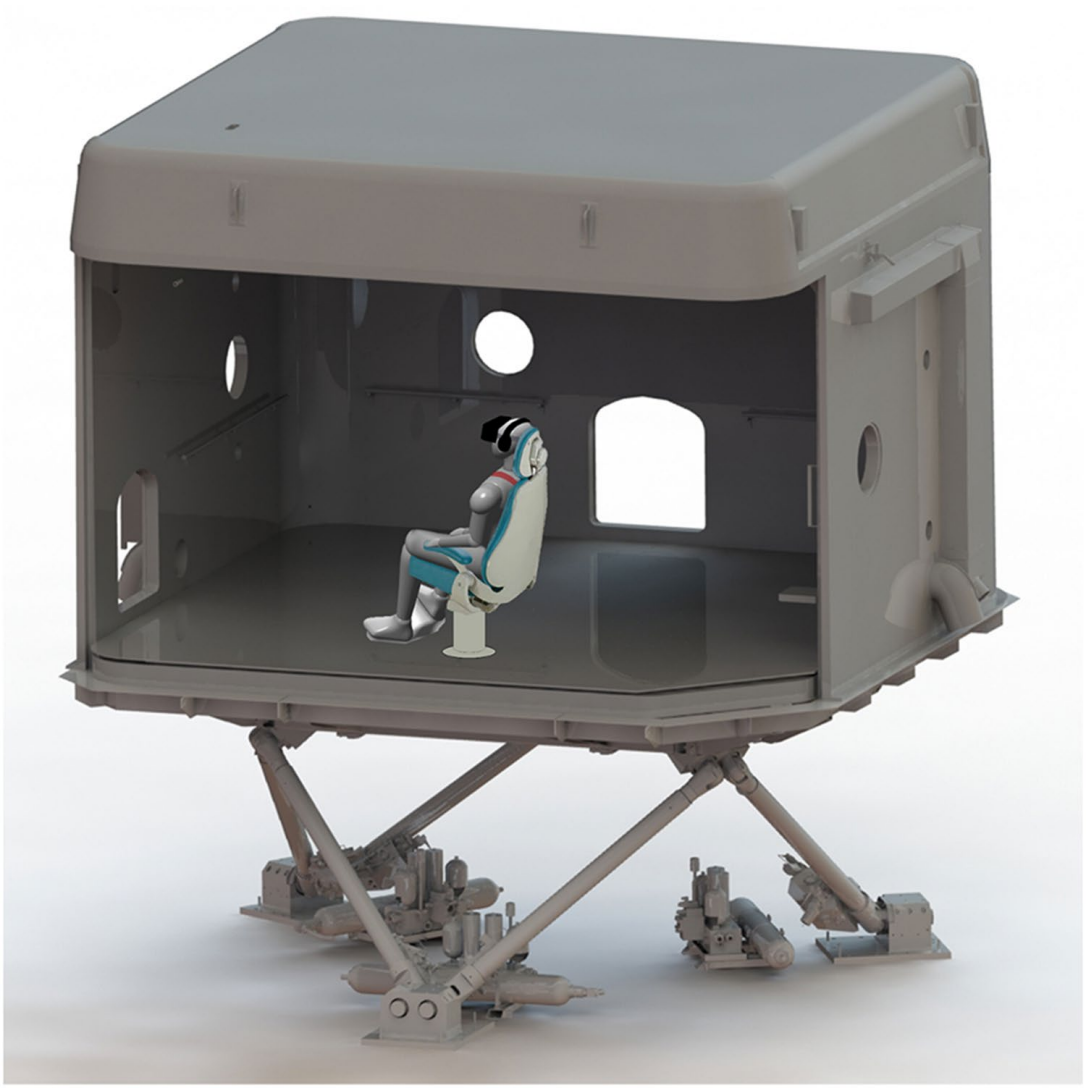
Vestibular psychophysics setup. Schematic of the laboratory setup for the psychophysical task including the 6-degrees-of-freedom motion platform.

The vestibular psychophysics tasks were performed within the KITE—Toronto Rehabilitation Institute’s Challenging Environment Assessment Laboratory (CEAL). CEAL contains a 6.0 m × 5.6 m × 4.1 m enclosed laboratory mounted on a 6-degrees-of-freedom hexapod motion base (i.e., capable of moving in all linear directions and rotating around pitch, yaw, and roll axes), with 60″ actuator arms allowing tilting up to 100 °/s^2^ in the pitch axis, and 8 m/s^2^ in the heave direction (see Fig. 1).

For this study, the laboratory was outfitted with a specially constructed chair designed to minimize participants’ head and body movements. The chair was cushioned with foam to reduce vibrotactile feedback. Participants were secured in their seats by means of a four-point harness and had their feet resting on foam mats at the base of the chair to restrict leg-movement and to also reduce vibrotactile cues to the feet during the task. A neck pillow was used to further limit proprioceptive feedback through incidental movement of the head or neck. Finally, participants were blindfolded and wore noise-cancelling headphones that presented white-noise throughout each block to limit the sound created by the hydraulics of the motion base. Lights were also dimmed inside the lab for the duration of the experiment. The experimenter sat inside the lab with the participants but communicated with them through a microphone feeding into the headphones. The goal of this setup was to reduce as many sensory cues to motion as possible to isolate information about the passive movements to those arising primarily from the vestibular system.

##### Movement specifications

There were four conditions in the main psychophysical task: heave detection, heave discrimination, pitch detection, and pitch discrimination. The point of rotation for pitch movements was at the approximate center of the head. Each trial consisted of (1) a standard movement and (2) a comparison movement. For detection, the platform remained stationary during the “standard movement” (see Table 2 for movement specifications). Magnitudes are stated as peak accelerations for both heave (m/s^2^) and pitch (°/s^2^) motions.

**Table 2:** Initial peak accelerations used for the psychophysical tasks.

Movement type	Detection task	Discrimination task
Heave	Standard movement = 0 m/s^2^ Initial comparison movement = 0.5 m/s^2^	Standard movement = 1.0 m/s^2^ Initial comparison movement = 1.5 m/s^2^
Pitch	Standard movement = 0 °/s^2^ Initial comparison movement = 3 °/s^2^	Standard movement = 20 °/s^2^ Initial comparison movement = 26 °/s^2^

The initial comparison movements in this table represent the initial acceleration values. These values changed throughout the session as a function of the PEST (*Parametric Estimation by Sequential Testing*) procedure outlined in the text.

**Figure 2:**
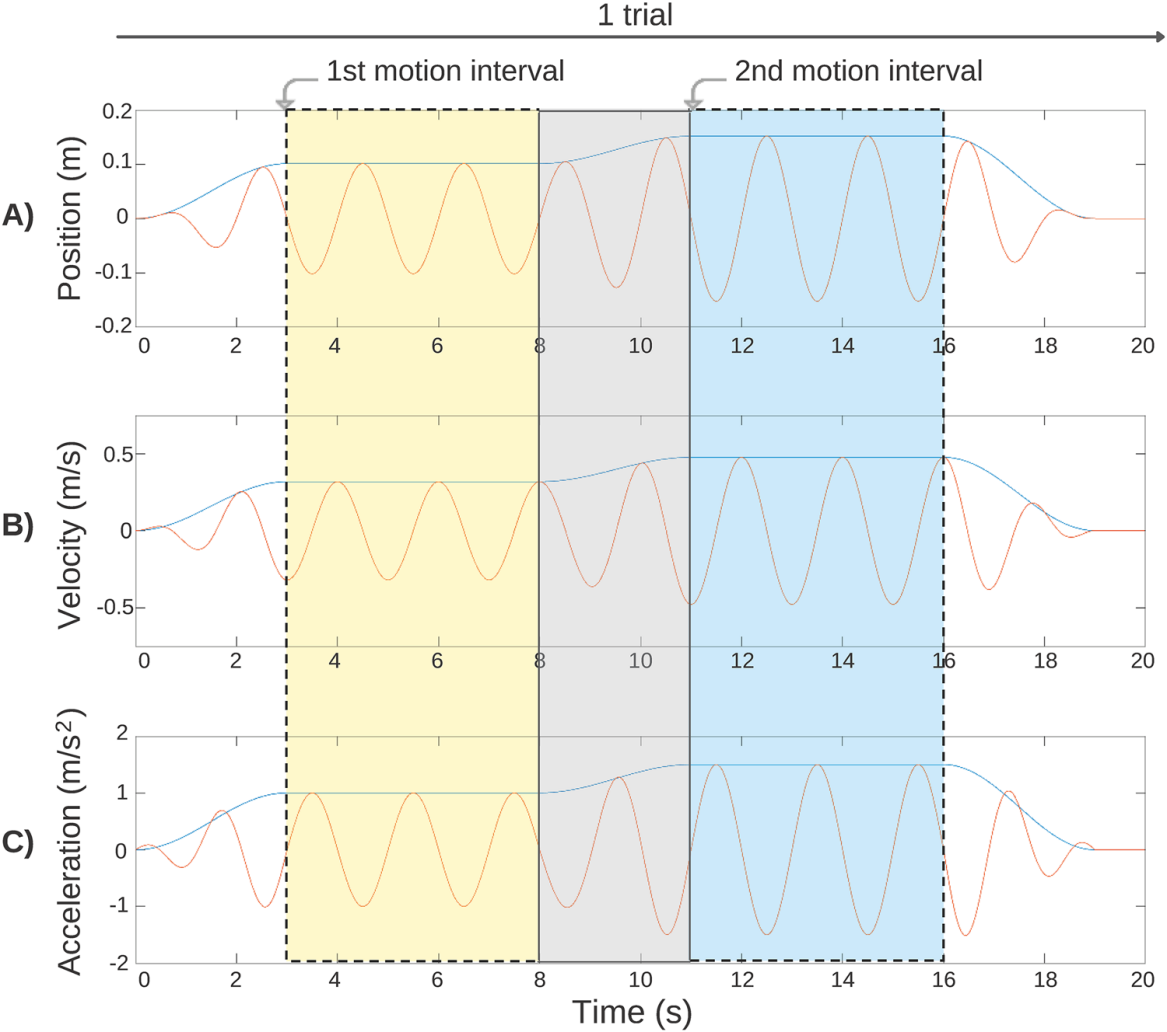
Diagrammatic representation of a single heave discrimination trial. (**A**) the position (m) relative to the upright start position, (**B**) the velocity (m/s) and (**C**) the acceleration (m/s^2^) of the motion base. For pitch trials, displacement was measured in degrees, velocity in °/s, and acceleration in °/s^2^. The yellow shaded area highlights the first movement (5 s; here, standard movement), the grey shaded area represents the fade-in between the first and second movement (3 s), and the blue region represents the second movement (5 s; here, comparison movement). The unshaded white regions represent the fade from no motion to the first movement (3 s), or from the second movement to no motion (3 s), and rest (1 s).

Each full trial consisted of a standard movement and a comparison movement presented in a random order^101^. The movements all followed the same profile. The platform was oscillated at 0.5 Hz either in pitch or heave, beginning at rest in a central position with the participant sitting upright. The platform was then oscillated sinusoidally around this position with a peak velocity that increased along a raised cosine velocity envelope reaching the desired value after three seconds. The platform then oscillated with this peak velocity, and the corresponding peak accelerations, for five seconds (Fig. 2, yellow shaded area). The peak velocity then changed in magnitude along a raised cosine velocity envelope for three seconds (Fig. 2, grey shaded area) and then oscillated with a second peak velocity and acceleration for another five seconds (i.e., the second movement of the trial; Fig. 2, blue shaded), before returning back to rest for three seconds. The motion base then rested for one second, or until the participant made their response. Each complete trial lasted approximately 20 s (see Fig. 2 for an example of a full heave discrimination trial).

##### Procedures

Participants completed both a two-interval detection task and a two-interval magnitude discrimination task for each of the two motion types—pitch and heave—resulting in four psychophysical conditions in total. The four conditions (heave detection, heave discrimination, pitch detection, pitch discrimination) were presented in a random order across participants to protect against effects of carryover, practice, or fatigue across conditions. Breaks were provided on an as-needed basis. In total, these four conditions took approximately one hour per participant to complete. Participants also completed a posturography task (described below) to assess their standing balance immediately before completing the vestibular perceptual psychophysics task.

**Detection** Each individual trial in the detection condition was comprised of two intervals: (1) a motion interval (in pitch or heave, depending on the condition) and (2) a no motion interval. The order of these two motion intervals were randomized across trials within a condition. After both intervals were presented (demarcated by a spoken “one” or “two”, via the headphones, at the point of the peak acceleration), participants were asked to state out loud which of the two intervals was the one in which they had moved (“one” or “two”). The acceleration of the motion presented was varied using a *Parametric Estimation by Sequential Testing* procedure (PEST^102^) until the participant’s detection threshold was reached. PEST is an adaptive staircase procedure that uses a set of rules to converge on perceptual thresholds corresponding quickly and efficiently to where participants were 70.7% correct, in this instance^103^. To vary the values presented logarithmically, the base-10 logarithms of the acceleration values (beginning with the initial comparison movement value; see Table 2) were used by the PEST and the PEST’s log output was exponentiated into peak acceleration values before being fed to the platform’s motors.

Using the logged acceleration values, the PEST decreased the magnitude of the subsequent trial by a single “step” (defined below) if the participant responded correctly on two consecutive trials. If they responded incorrectly once, the value would increase by one step. The initial step sizes were log (0.1) for the heave condition and log (0.2) for the pitch condition. On the third step in the same direction, the size of the step doubled. This was unless the third step in the same direction was before the last reversal, in which case the rule was to wait until the fourth step. A change in direction (e.g., going from an increase to a decrease in peak acceleration, or step) represented a “reversal”. Eight reversals or 60 trials, whichever came first, signaled the end of a block of trials (i.e., vestibular threshold reached). The 70.7% correct threshold was calculated by averaging the accelerations of the last three reversals^103^.

**Discrimination** As in the detection condition, the discrimination condition used a similar PEST procedure to determine participants’ movement discrimination thresholds, except with the PEST being applied to the delta relative to the standard movement rather than the comparison amplitude. Participants were required to discriminate (i.e., report which motion felt larger) between two sequentially presented movements of different magnitudes: (1) a standard movement in pitch or heave and (2) a comparison movement, also in pitch or heave. These two movement intervals were separated by a sound file comprised of a spoken “one” or “two” presented through their headphones. Peak accelerations of the comparison movement interval were determined via the above-described PEST procedure using the same initial step sizes and the same termination criteria. The 70.7% correct discrimination thresholds were calculated by averaging the accelerations of the last three reversals^103^.

### Posturography task

Participants also completed a posturography task to assess their standing balance. In this task, participants stood in parallel pose (i.e., feet facing forward, approximately 8″ apart) for 30 s^104^ on a forceplate (AMTI MSA-6 MiniAmp strain gage amplifier) which captured their center of pressure (COP) path length (cm) and velocity (cm/s). Signals from the forceplate were collected at a sampling rate of 1000 Hz. This was completed for four different trial types: (1) eyes open standing directly on the forceplate (EOF; “firm surface”), (2) eyes open on a piece of high-density foam placed on the forceplate (EOC; “compliant surface”; AIREX, Balance-Pad; 50 × 41 × 6 cm; density = 55 kg/m^2^), (3) eyes closed on a compliant surface (ECC), (4) eyes closed on a compliant surface while wearing noise-cancelling headphones for sound suppression (ECSS). Participants wore a loose harness during the procedure to protect against falls.

Once collected, the first five seconds of the data were discarded^104^. The remaining data were passed through a 2nd order zero-lag dual-pass Butterworth filter with a 6 Hz cut-off frequency. Mean COP path lengths, velocity, and velocity root-mean-square (RMS)^105–108^ were extracted from the data in MATLAB for each of the four trial types (recorded and analyzed separately). COP path length was defined as the absolute length of sway in centimeters produced by the participant during each of the conditions. Increased postural sway was therefore associated with greater COP path lengths. Measures of velocity were obtained by taking the COP excursion and dividing it by trial time, with poorer postural control being related to larger COP velocity. To obtain velocity RMS, the square root of the mean of the squares of the velocity measures were computed. Greater velocity RMS was related to more variable postural sway.

### Data analysis

All analyses were run using the threshold values obtained above in R 3.6.0^109^. All data were winsorized to treat potential outliers using the “DescTools” R package^110^. If the data are not winsorized the same results are significant. The data were then evaluated for skewness using the “e1071” package^111^ and evaluated for normality using a generalized Shapiro–Wilk test for normality, then log-transformed to meet the Gaussian assumption (although in-text means and standard deviation are calculated using raw, winsorized data to facilitate comparisons in the literature). To compare the older and younger adults’ vestibular perceptual thresholds in each of the four vestibular perceptual tasks (heave and pitch detection and discrimination) a series of four independent sample *t*-tests were conducted. Note that the deltas of the discrimination thresholds are reported here (e.g., instead of 22.6 m/s^2^, we report the delta: 2.6 m/s^2^). We also calculate and report effect sizes (Cohen’s *d*). Next, to compare COP path length, velocity, and velocity-RMS across the four posturography tasks, between older and younger adults we ran three separate 2 (Age Group: younger, older) $\times$ 4 (Condition: EOF, EOC, ECC, ECSS) mixed factorial ANOVA. As with the threshold data, the posturography data were winsorized to treat potential outliers using the “DescTools” R package^110^ and evaluated for normality using a generalized Shapiro–Wilk test for normality, then square-root transformed to meet the Gaussian assumption. Note that one younger adult participant’s data were not collected due to technical difficulties. Tukey-corrected post-hoc *t*-tests were used to explore significant interaction effects. Greenhouse–Geisser corrections were applied to correct for violations of sphericity.

We also conducted a series of Bonferroni-corrected Pearson correlations on the raw (i.e., not log-transformed) winsorized data to assess the extent to which COP path length (from each of the four posturography conditions) are associated with each of the four vestibular perceptual thresholds. If the data are not winsorized the same correlations are significant, although the associations are stronger in the older adults and the ECSS condition becomes significantly positively associated with the Heave Detection condition (*r* = 0.47; *p* = 0.04). These correlations were conducted separately for the older adult participant group and the younger adult participant group, to compare whether these associations differed as a function of age.

## Results

### Vestibular psychophysics task

#### Detection task

**Figure 3:**
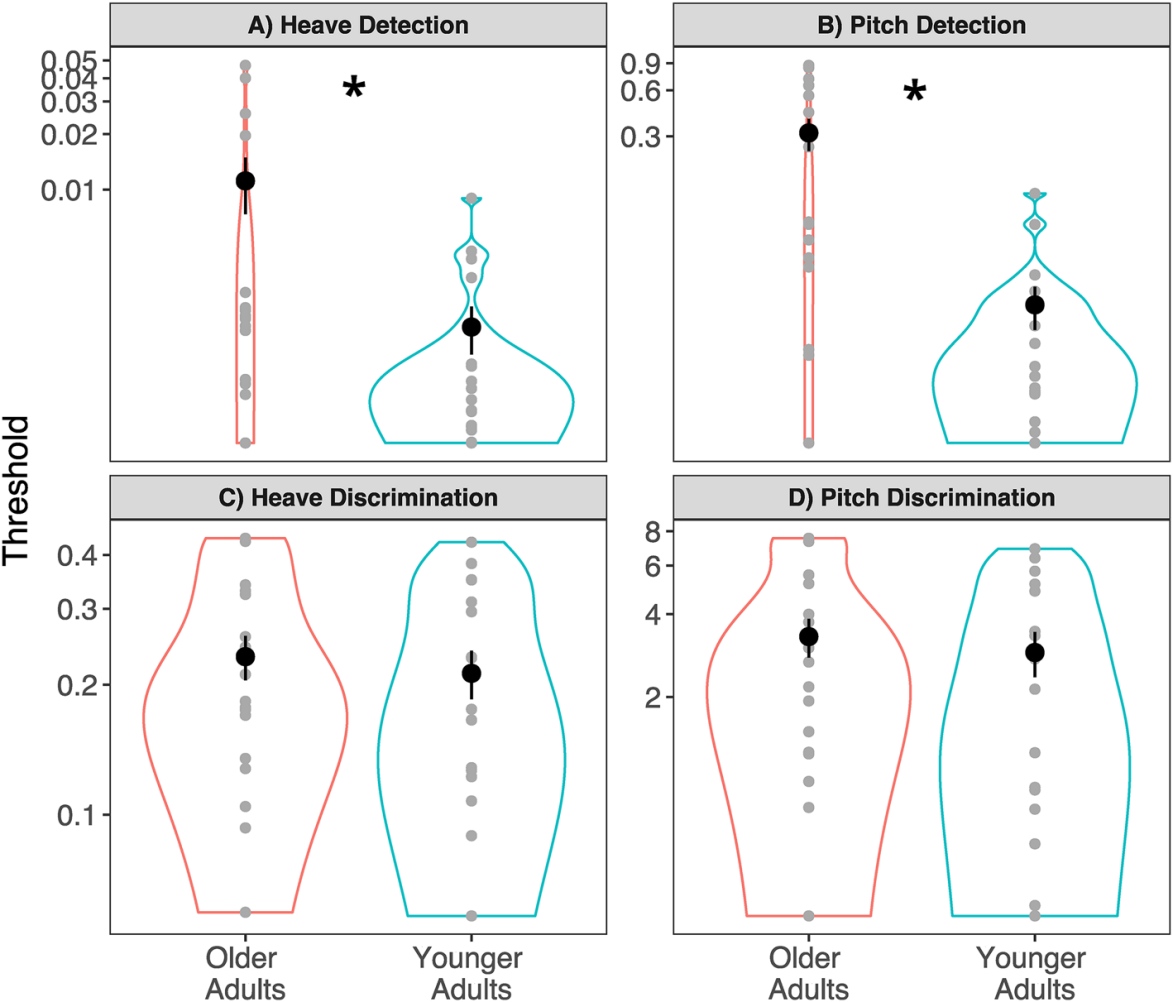
Heave and pitch detection and discrimination thresholds. *p < 0.05. Graphs show data from the, (**A**) Heave Detection condition, (**B**) Pitch Detection condition, (**C**) Heave Discrimination condition, and (**D**) Pitch Discrimination condition. All data are plotted on logarithmic scales. Thresholds for heave data are in m/s^2^, and for pitch data in °/s^2^. Small points represent individual data. The larger black points represent the group means and error bars are standard errors. The width of the borders of the violin plot represents frequency count at each value on the y-axis.

Independent sample *t*-tests revealed that older adults had significantly greater heave detection thresholds (*M* = 0.022 m/s^2^, *SD* = 0.051) than younger adults (*M* = 0.002 m/s^2^, *SD* = 0.002, *t*(34.98) = − 2.89, *p* = 0.007, *d* = 0.554) (Fig. 3A). Further, older adults had significantly greater pitch detection thresholds (*M* = 0.329 °/s^2^, *SD* = 0.355, *d* = 1.210) than younger adults (*M* = 0.024 °/s^2^, *SD* = 0.032, *t*(30.51) = − 4.95, *p* < 0.001) (Fig. 3B).

#### Discrimination task

Independent sample *t*-tests showed that older adults (*M* = 0.241 m/s^2^, *SD* = 0.137) and younger adults (*M* = 0.211 m/s^2^, *SD* = 0.119) did not have significantly different heave discrimination thresholds (*t*(34.68) = − 0.61, *p* = 0.549, *d* = 0.234) when discriminating from a baseline of 1m/s^2^ (Fig. 3C). Likewise, there were no significant differences between pitch discrimination thresholds for older (*M* = 3.41 °/s^2^, *SD* = 2.53) and younger adults (*M* = 2.89 °/s^2^, *SD* = 2.25, *t*(31.23) = − 0.783, *p* = 0.439, *d* = 0.217) when discriminating from a baseline of 20°/s^2^ (Fig. 3D).

### Posturography

**Figure 4:**
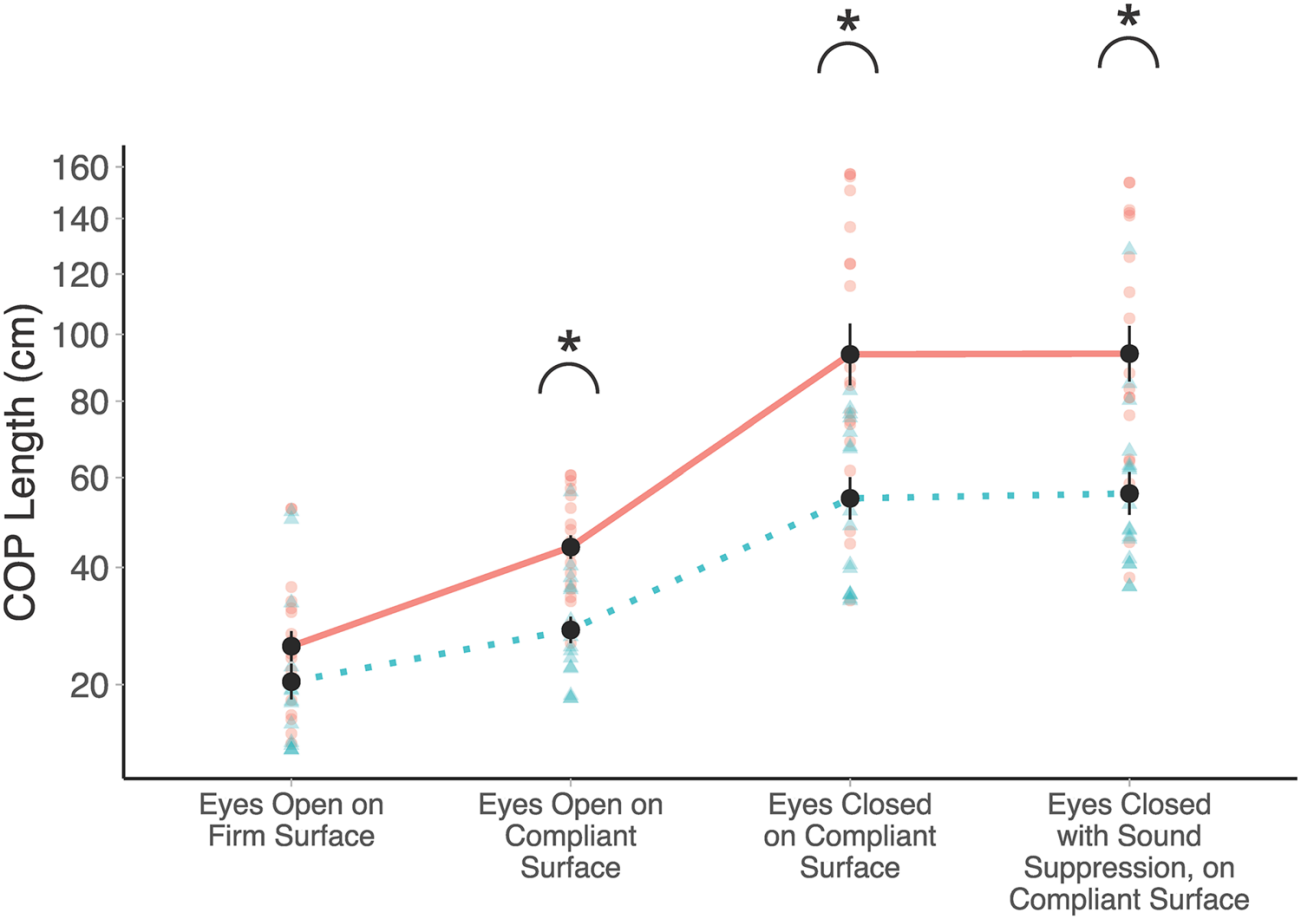
Posturography data for older and younger adults across all four conditions. *p < 0.05. Mean COP path length (cm) are plotted (older adults = solid red, younger adults = dotted blue). Individual participant data are plotted using single points (older adults = red circles, younger adults = blue triangles). Data are plotted on a square-root scale.

A 2 (Age Group) $\times$ 4 (Condition: EOF, EOC, ECC, ECSS) mixed factorial ANOVA, on COP path length, revealed a significant main effect of Age Group, *F*(1, 34) = 14.83, *p* < 0.001, indicating that older adults (*M* = 46.8 cm, *SD* = 33.2) had significantly greater COP path lengths than younger adults (*M* = 28.1 cm, *SD* = 17.6) overall. The COP path lengths are shown for all groups in Fig. 4. There was also a significant main effect of Condition, *F*(1.59, 54.15) = 105.24, *p* < 0.001, showing significant differences between all conditions (*p* < 0.001), with the exception of ECC relative to ECSS, *t*(102) = − 0.266, *p* = 0.993. Specifically, easier posturography conditions (e.g., EOC) showed smaller COP path lengths than more difficult posturography conditions (e.g., ECC). There was also a significant Age Group $\times$ Condition interaction (*F*(1.59, 54.15) = 5.50 *p* = 0.011). Post-hoc *t*-tests showed that older adults had significantly greater COP lengths relative to younger adults in the three hardest conditions: EOC (*t*(72,8) = − 2.452, *p* = 0.017), ECC (*t*(72.8) = − 3.958, *p* < 0.001), and ECSS (*t*(72.8) = − 4.818, *p* < 0.001), but not the EOF condition (*p* > 0.05).

These analyses were repeated with COP velocity (m/s), as well as COP velocity root-mean-square (RMS). Similar results were observed (see Supplementary Information S.2, for full statistical analyses), namely that COP velocity and velocity-RMS were larger in older adults relative to younger adults, with similar differences between the conditions. Likewise, these analyses revealed the same Group $\times$ Condition interaction, indicating greater velocity as well as velocity-RMS in the three hardest conditions (EOC, ECC, and ECSS) for older adults relative to younger adults.

### Correlational analyses between vestibular thresholds and posturography

**Figure 5:**
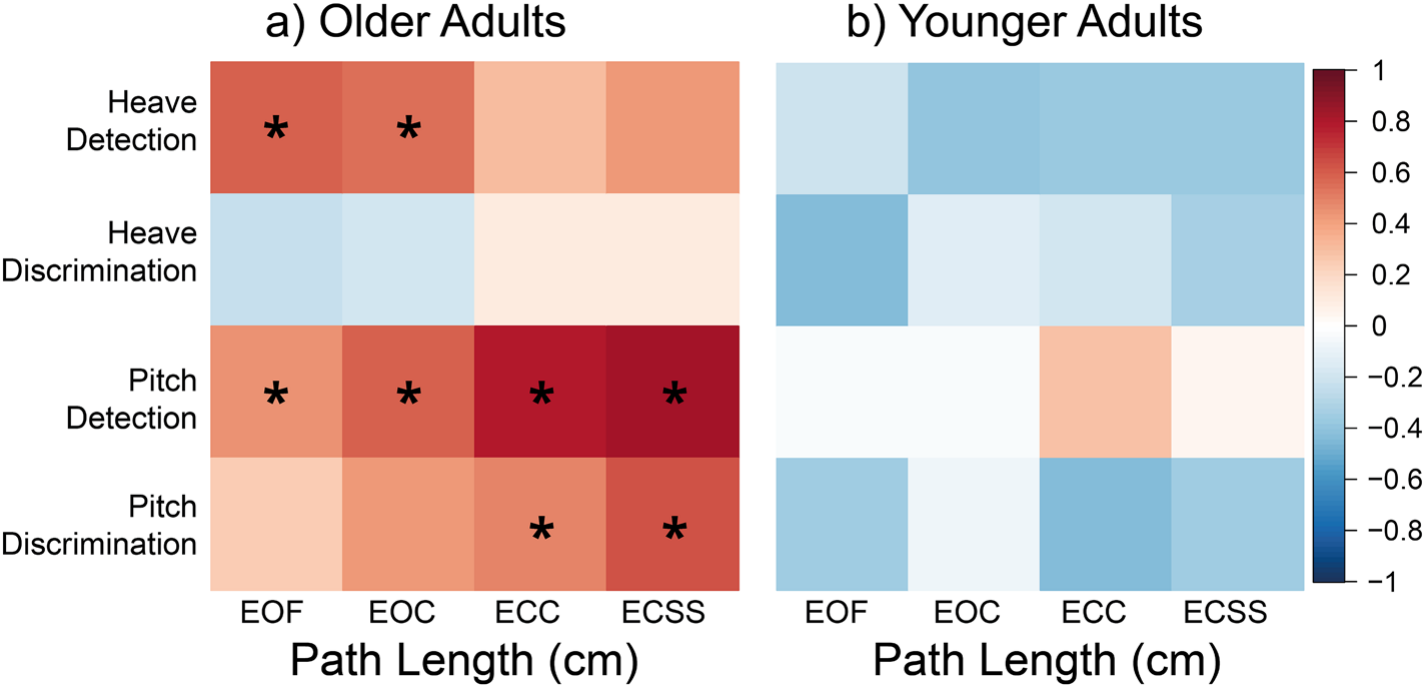
Correlation heatmaps. Graph illustrating the associations between posturography measures (i.e., COP path length, cm) and perceptual thresholds (i.e., m/s^2^ for heave, and °/s^2^ for pitch) in, (**a**) older adults, and (**b**) younger adults. Blue squares represent negative correlations and red squares represent positive correlations. Lighter squares represent weaker correlations and darker squares represent stronger correlations. Correlations were Bonferroni-corrected. *p < 0.05. *EOF* Eyes open, firm surface, *EOC* eyes open, compliant surface, *ECC* eyes closed, compliant surface, *ECSS* eyes closed, with sound suppression on a compliant surface.

A series of Bonferroni-corrected Pearson correlations were used to examine the relationship between the four postural task measures for COP path length and the four vestibular perceptual threshold measures in older adults (Fig. 5a) and younger adults (Fig. 5b). Please see the Supplementary Information S.3 for scatterplots, as well as Fisher’s r-to-Z transformation to compare the younger adult and older adults’ correlations. Additional, albeit not significant, Bonferroni-corrected correlations between the four psychophysical measures can be found in the Supplementary Information S.4.

#### Perceptual thresholds and posturography

In older adults, heave detection thresholds were positively correlated with COP path length for EOF (*r* = 0.60, *p* < 0.007) and EOC (*r* = 0.56, *p* = 0.013) conditions, such that higher detection thresholds were associated with greater postural sway (Fig. 5a). Likewise, older adults’ pitch detection thresholds were positively correlated with COP path length for EOF (*r* = 0.46, *p* < 0.048), EOC (*r* = 0.59, *p* = 0.008), ECC (*r* = 0.78, *p* < 0.001) and ECSS (*r* = 0.82, *p* < 0.001) conditions. Finally, older adults’ pitch discrimination thresholds were positively associated with COP path length for the ECC (*r* = 0.49, *p* < 0.033) and ECSS conditions (*r* = 0.64, *p* = 0.004), but there were no correlations between heave discrimination and any measures of COP path length. For younger adults, there were no significant associations observed between any of the posturography measures and vestibular perceptual thresholds (Fig. 5b). We did, however, observe trending negative correlations, similar to ^79^ who also found trending negative associations between postural sway and rotation direction-discrimination thresholds in younger adults.

In order to account for the continuous effect of age independently influencing postural sway, we also ran multivariate analyses to predict postural sway from the vestibular perceptual thresholds and age (see Supplementary Information S.5). These results show that higher heave detection thresholds significantly predicted larger postural sway in the EOF (*p* < 0.001) and EOC (*p* = 0.009) conditions, but that pitch detection predicted ECC (*p* < 0.001) and ECSS (*p* < 0.001).

## Discussion

We examined age-related changes to vestibular perceptual thresholds for heave and pitch motions. We also explored whether quiet standing balance performance was associated with vestibular perceptual thresholds in older or younger adults. We found that, compared to younger adults, older adults showed significantly higher vestibular detection thresholds in heave, and reported for the first time to our knowledge, in pitch. There were similar patterns of non-significant age-related effects for the heave and pitch discrimination task thresholds, which may be a result of less robust age-related effects and/or insufficient power to observe these effects. Therefore, these results may be a conservative estimation of age-related changes in vestibular thresholds across different motion types and motion tasks.

We also found that older adults had significantly greater COP path lengths relative to younger adults in all conditions except for the easiest condition (i.e., standing on a firm surface with eyes open). Importantly, we also found that, for older adults, but not younger adults, measures of postural sway (COP path length) were positively associated with vestibular perceptual thresholds, such that poorer balance (greater sway) was associated with higher heave and pitch detection thresholds.

### Age-related changes in pitch and heave perception

#### Vestibular detection task

Older adults had higher heave and pitch detection thresholds than younger adults. These results are consistent with current studies in the literature that have also shown older age to be associated with increased direction discrimination (i.e., vestibular perceptual) thresholds in the heave direction^15,16,70^, as well as in surge, sway, and roll-tilt^15,16,18,19,70^, but not yaw rotation^16,70^ (but see ^17^ who found no age differences in yaw motion perception during a two-interval detection and two-interval magnitude discrimination task). Our study is the first to our knowledge to also examine age-related differences in pitch perception in older and younger adults. Together these results suggest that aging is associated with declines in a range of vestibular perceptual sensitivities across different vestibular perception tasks and movement axes.

Histological, microscopic, and clinical assessments of vestibular functioning have long associated the aging process with vestibular end-organ deterioration. For instance, aging has been linked with significant declines in the number of hair cells, degeneration of the vestibular ganglion, deterioration of the otoconia of the otoliths (see ^5,6,27^ for reviews), and reduced oVEMP and cVEMP responses^7,38,97,99^. There is also some evidence of age-related decreases in vestibular cortical network connectivity^112^, although other studies have shown age-related central gains in vestibular perception, which seem to at least partially compensate for peripheral declines in end-organ responsiveness^113^.

Despite reporting no diagnosed vestibular impairments or chronic dizziness, some older participants in this study showed evidence of end organ dysfunction of the otoliths (VEMPs), particularly the utricle (oVEMP), which is consistent with prior research^5,7,99,114,115^. This may have contributed to older adults’ reduced ability to detect pitch and heave motions. It is important to note, however, that the recently described diagnostic criteria for presbyvestibulopathy (PVP; age-related vestibular loss) includes measures of VOR gain (e.g., vHIT measures), but does not include VEMP responses^84^. It was concluded that, because an absence of VEMP responses is frequently observed in older adults, the significance of these absences for diagnostic purposes is not well understood. In our study, presence, or absence of a VEMP response was not significantly correlated with heave or pitch detection thresholds in our participants (Supplementary Table S.6.1, Supplementary Materials), except for cVEMP presence being associated with reduced heave detection thresholds but only when not correcting for multiple comparisons, (*r* = − 0.55, *p* = 0.042). Likewise, we did not observe a significant correlation between VEMP presence and COP path length (Supplementary Table S.6.2, Supplementary Materials). These results are in line with previous findings which showed no associations between cVEMP or oVEMP amplitudes, and vestibular perceptual thresholds in the heave direction, in healthy controls and patients with bilateral vestibulopathy (BV)^116^. Therefore, the extent to which VEMP responses might relate to observed age-related differences in vestibular perceptual thresholds remains unclear.

#### Vestibular discrimination task

While we observed age-related differences in pitch and heave detection thresholds, no age-related differences were observed for pitch or heave discrimination tasks. This suggests that while older adults might have a preserved ability to differentiate between two motions of similar magnitudes, their sensitivity to detecting these same motions may be reduced relative to younger adults.

With regards to *direction* discrimination (which differs from this study’s magnitude discrimination task), previous research has found that older adults tend to demonstrate larger thresholds than younger adults^15,16,18,19,70^. We suggest a number of possible reasons for this discrepancy.

First, the nature of the task used in this study (magnitude discrimination) is different than those used in the previous aging literature (direction discrimination) where age-related differences were found^15,16,18,19,70^. To our knowledge, only Chang and colleagues investigated magnitude discrimination in older and younger adults, and also found no significant differences^17^. Furthermore, these previous direction-discrimination tasks used a one-interval forced choice task, whereas ours used a two-interval forced-choice task, which maybe have further influenced threshold values. For instance, two-interval detection or discrimination tasks such as those used in this current study, are associated with thresholds that are $1/\sqrt{2}$ times smaller than 1-interval detection or discrimination tasks^103^. Such differences in magnitude may have had an impact on the overall thresholds obtained by participants, leading to the null results observed in this condition.

Secondly, the frequency of the motion presented during vestibular perception tasks has been found to influence whether age differences are observed. For instance, Roditi and Crane^70^ found that relative to younger adults, older adults had poorer surge direction-discrimination thresholds if motions were presented at 0.5 Hz, but not if presented at 1 Hz. Indeed, previous studies have found that regardless of age, participants tend to show higher thresholds for lower motion frequencies^101,117,118^. Once again, smaller thresholds obtained in both groups may have eliminated such previously observed age-related differences.

Finally, the type of motion profile (e.g., single sine waves versus sinusoidal oscillation), may also affect threshold values and whether age-related differences are observed. Repeated sine wave procedures offer a greater number of samples per trial on which to base perceptual estimates, thereby leading to greater sensitivity (Fig. 2). For example, Chang and colleagues^17^ and Bermùdez Rey and colleagues^16^ evaluated magnitude discrimination (using repeated sine waves) and direction discrimination (using a single sine wave) respectively, in older adults, for yaw rotation. Chang and colleagues’^17^ older adults showed thresholds which were almost half (0.81 ($\pm$ 0.42) °/s) those obtained by Bermùdez Rey and colleagues (^16^; 1.45 (range 1.14–1.84) °/s).

Ultimately, future studies should more systematically evaluate how different frequencies, motion profiles, and tasks influence the extent to which age-related differences in vestibular perception are observed.

### Associations between vestibular perceptual thresholds and postural stability in older and younger adults

Our older adults were less stable than younger adults in all but the easiest (i.e., standing on a firm surface with eyes open) balance conditions. Such age-related differences were observed consistently, regardless of whether we examined COP path length, velocity, or velocity-RMS. These results are consistent with previous literature showing that older adults demonstrate greater COP path length, velocity, and variability compared to younger adults, especially when multiple senses are impoverished or challenged (e.g., vision and proprioception^9–11,119^. Postural control generally relies on the contributions and integration of visual, vestibular, and somatosensory cues^119–122^. This process of multisensory integration during standing balance is particularly relevant in the context of aging since older adults tend to show heightened multisensory integration relative to younger adults^20,123–127^ and may weigh less reliable sensory inputs more than is optimal^20,128^.

A novel finding of the present study is that poorer vestibular perceptual sensitivity (i.e., higher thresholds) was associated with greater postural sway, but only in older adults. These associations were found particularly for pitch and heave detection (conditions which showed significant age-related effects in the psychophysical tasks), as well as pitch discrimination. Interestingly, for pitch detection and discrimination, the magnitude of the correlations increased systematically as the difficulty of the postural task also increased. One possible reason for this linear association is that the more difficult postural tasks also increase the need to rely on vestibular input for balance maintenance (e.g., in the absence, or limited presence, of other sensory cues). Specifically, standing on a compliant surface reduces the reliability of somatosensory cues, closing the eyes eliminates the availability of visual cues, and auditory cues are reduced with sound suppression. As such, age-related declines in vestibular perceptual sensitivity may be increasingly consequential to posture when other sensory inputs become more impoverished. Importantly, we found fewer significant associations between postural stability and vestibular discrimination thresholds. This lack of association may be due to the nature of the posturography task (i.e., quiet standing). Specifically, given that precise discrimination estimates may be especially important for *responding* to changes in balance/posture (as opposed to simply maintaining stable balance/posture), using dynamic posturography tasks (e.g., recovery from perturbation) in future may result in even stronger and more consistent associations.

With regards to the association between postural task difficulty and pitch detection *and* discrimination, a possible explanation for this increased correlation with age might be related to changes in vestibular end organ functioning with age. For instance, semicircular canals—necessary for pitch detection—show more age-related dysfunction in clinical tests than the utricle or saccule^38^, as well as a more pronounced loss of Type I hair cells^29,32,33^ and greater degeneration of the superior (relative to the inferior or posterior) vestibular nerve^36^. Such changes might reflect the extent to which pitch sensitivity contributes to postural stability (e.g., COP path length) as reliance on vestibular information during standing balance increases. Furthermore, it has been postulated that changes in end organ functioning may be associated with increased neural noise^18,19,129^ resulting in higher perceptual thresholds^130^ and greater postural sway^131–133^.

Importantly, no correlations between standing balance and vestibular perception were observed in the younger adults. Future studies should examine whether vestibular perceptual thresholds along other movement axes might be associated with postural sway in younger and older adults. For instance, higher roll-tilt (at 0.2 Hz) vestibular perceptual thresholds have been found to be significantly associated with failing the hardest condition of a modified Romberg balance test, regardless of age^18^ and previous studies have found lateral translation vestibular thresholds to be significantly associated with postural sway in younger adults^79^.

### Limitations and future directions

#### Participant sample

Participants in this study underwent rigorous screening, which allowed us to control for certain age-related factors, including for example, cognitive decline^133–139^, age-related hearing loss and tinnitus^85,88,90,98,140^, diagnosed vestibular disorders^141^, and other serious health conditions. However, these common age-related conditions are likely to also influence vestibular perception and postural control and, as such, the sample in this study may not be representative of the typical older adult population. Instead, these results may be a conservative estimate of age-related changes to vestibular perception. Future studies can help disambiguate age-related changes in vestibular perceptual sensitivity thresholds in a more representative sample of older adults by collecting data from participants with a range of sensory, motor, and cognitive abilities. Furthermore, they can also consider collecting additional baseline sensory, cognitive, and motor measures from both samples of participants (older and younger) to determine whether there are associations among these measures and experimentally test outcomes across the lifespan.

#### Extra-Vestibular cues

It is also important to acknowledge the potential effect of extra-vestibular cues on participants’ detection and discrimination thresholds. Specifically, this study was designed to reduce the influence of non-vestibular cues such as visual (blindfolds and dimmed lights), auditory (white noise, passive noise-suppressing headphones), proprioceptive (four-point harness, inflatable neck-pillow), and vibrotactile (padded seating and foam footrest)—it remains possible that subtle vibration or proprioceptive cues may have still facilitated perceptual judgements during the vestibular psychophysical tasks. Access to these additional non-vestibular sensory cues may account for the very low perceptual thresholds we obtained, particularly in the detection task—although some of these cues (e.g., vibration) could have also been detected by the vestibular system. In fact, previous research has shown that detection thresholds are as much as 31 × smaller than direction-discrimination thresholds^142^. Such differences in thresholds have been suggested to be due to the influence of vibrotactile cues during detection tasks^142^. Future studies could consider adding additional vibrational masking noise during the tasks to further control for such additional cues and better account for this variability in our participants.

#### End-organ testing

In this study, we expected that older adults might demonstrate some evidence of vestibular end organ dysfunction^5–7,38,99,114,115^ despite the absence of a clinically diagnosed vestibular disorder^84^. To quantify such dysfunctions, we measured canal and otolith functioning using VEMP and vHIT measures that included a particular set of testing parameters (e.g., VEMP frequency of 500 Hz), and further correlated these metrics to measures of COP and vestibular thresholds. The lack of correlations of vestibular thresholds with vHIT or VEMP suggests that common age-related declines in vestibular perceptual thresholds are not due solely to end organ or afferent deficits (see ^84^ for a discussion on the lack of association between measures of end organ functioning and presbyvestibulopathy). However, this choice of parameters may affect whether age-related differences are observed. While for many older adults, VEMPs evoked at stimulus frequencies of 500 Hz show the greatest response, many other older adults will only show evoked oVEMP or cVEMP responses at higher tone burst frequencies (e.g., 750 Hz or 1000 Hz). Future studies should more carefully evaluate the associations between vHIT and VEMP measures and vestibular perceptual thresholds using a range of end organ testing parameters. Finally, future studies could also consider evaluating VEMP and vHIT responses in the younger adult participant group to better understand changes in the associations among end organ functioning, vestibular perception, and postural control across the lifespan.

## Conclusion

Older adults had higher heave and pitch detection thresholds than younger adults which, together with pitch discrimination thresholds, were associated with increased postural sway (i.e., COP path length) particularly when sensory conditions were impoverished. These results could have implications in the development of screening tools to detect mobility declines in older adults, given that measurable declines in vestibular perceptual sensitivity were associated with poorer postural stability. These convergent perceptual and behavioral measures may therefore allow for better identification of falls risk.

## Supplementary Information

Supplementary Information.
