# Supplementary material for: Age-related changes to vestibular heave and pitch perception and associations with postural control
Source: Sci Rep. 2022 Apr 19;12:6426. doi: 10.1038/s41598-022-09807-4 (PMC9018785; doi:10.1038/s41598-022-09807-4)
Supplement: Supplementary file 1 — Supplementary Information. [file 41598_2022_9807_MOESM1_ESM.docx]

# S.1 Detailed Methods

## End-Organ Testing

### Video Head Impulse Test (vHIT)

To perform the vHIT test, participants wore a pair of goggles equipped with an eye-tracking device (EyeSeeCam goggles by Interacoustics, Middlefart, DK) and were asked to maintain fixation on a laser point on the wall ahead of them (approx. 1.5m away) while the experimenter rotated their heads to the right or left (randomized, 20 impulses per side; head rotation: 15-20°, duration 150-200 ms, peak velocity 150-200 deg/s). Perfect canal functioning evokes counter-rotational eye-movements that directly mirror that of the imposed head-movements, resulting in a VOR gain of 1.0. A gain less than 0.7 suggests impaired semicircular canal functioning (Halmagyi et al., 2017; Janky et al., 2017; MacDougall et al., 2013; McGarvie et al., 2015), since impaired canal functioning reduces eye velocity relative to head velocity.

### Vestibular Evoked Myogenic Potentials (VEMP)

To evoke VEMP responses, short (4ms, at a rate of 5.1Hz), high-intensity (97 dB, 500 Hz) clicks were presented to the participant's ear via insert headphones, with VEMP responses collected using the Neuroscan Synamps 2 (Compumedics Neuroscan, El Paso, TX) in most participants, and through the Interacoustics Eclipse EP25 (Interacoustics, Denmark) in the remaining participants. Both oVEMPs and cVEMPs were reviewed by a registered clinical audiologist (M.H.) and one of the authors (G.A.G), and demonstrated high inter-rater reliability (88% agreement). Raters performed their review of the VEMPs separately, and blindly. Disagreements in ratings were resolved by using another trained author’s blind assessment of the VEMP (J.J.G.). Additional details on the VEMP and vHIT testing parameters can be found in Gabriel et al. (2021).

# S.2 Detailed Posturography Results

## Velocity

*Figure S.2.1. Velocity data for posturography task in older and younger adults across all four conditions.* p < .05*Mean COP velocity (cm) is plotted (older adults = solid red, younger adults = dotted blue). Individual participant data are plotted using single points (older adults = red circles, younger adults = blue triangles). Data are plotted on a square-root scale.

**Table S.2.1**

*ANOVA results of the Posturography Task for Measures of Velocity*

| Predictor | *t* | *df* | *p* | $\beta$ | *SE* | *F* | *df* | *p* |
| --- | --- | --- | --- | --- | --- | --- | --- | --- |
| Age Group |  |  |  |  |  | 23.35 | .257 | < .001 |
| Condition |  |  |  |  |  | 116.48 | .606 | < .001 |
| ECC - ECSS | -0.196 | 102 | .997 | -0.009 | 0.048 |  |  |  |
| ECC - EOC | 10.458 | 102 | < .001 | 0.504 | 0.048 |  |  |  |
| ECC - EOF | 14.992 | 102 | < .001 | 0.723 | 0.048 |  |  |  |
| ECSS - EOC | 10.654 | 102 | < .001 | 0.514 | 0.048 |  |  |  |
| ECSS - EOF | 15.188 | 102 | < .001 | 0.732 | 0.048 |  |  |  |
| EOC - EOF | 4.534 | 102 | < .001 | 0.219 | 0.048 |  |  |  |
| Age Group (Younger Adults - Older Adults) $\times$ Condition |  |  |  |  |  | 5.06 | 0.063 | .013 |
| EOF | -1.322 | 91.6 | .190 | -0.117 | 0.088 |  |  |  |
| EOC | -2.690 | 91.6 | .009 | -0.237 | 0.088 |  |  |  |
| ECC | -4.912 | 91.6 | < .001 | -0.433 | 0.088 |  |  |  |
| ECSS | -4.803 | 91.6 | < .001 | -0.424 | 0.088 |  |  |  |

*Note*: The dependent variable was Velocity. Paired *t*-tests were corrected with Tukey-method to control for familywise errors. Sphericity was corrected using Greenhouse-Geisser method.

EOF = Eyes-Open, Firm Surface; EOC = Eyes-Open, Compliant Surface; ECC = Eyes-Closed, Compliant Surface; ECSS = Eyes-Closed with Sound Suppression.

## Velocity Root Mean Square (RMS)

*Figure S.2.2. Velocity-RMS data for posturography task in older and younger adults across all four conditions.* p < .05*Mean COP velocity root mean square (RMS) (cm) is plotted (older adults = solid red, younger adults = dotted blue). Individual participant data are plotted using single points (older adults = red circles, younger adults = blue triangles). Data are plotted on a square-root scale.

**Table S.2.2**

*ANOVA results of the Posturography Task for Measures of Velocity-RMS*

| Predictor | *t* | *df* | *p* | $\beta$ | *SE* | *F* | *df* | *p* |
| --- | --- | --- | --- | --- | --- | --- | --- | --- |
| Age Group |  |  |  |  |  | 19.93 | .244 | < .001 |
| Condition |  |  |  |  |  | 113.36 | .600 | < .001 |
| ECC - ECSS | -0.304 | 102 | .990 | -0.017 | 0.056 |  |  |  |
| ECC - EOC | 10.121 | 102 | < .001 | 0.561 | 0.056 |  |  |  |
| ECC - EOF | 14.798 | 102 | < .001 | 0.821 | 0.056 |  |  |  |
| ECSS - EOC | 10.426 | 102 | < .001 | 0.578 | 0.056 |  |  |  |
| ECSS - EOF | 15.103 | 102 | < .001 | 0.8375 | 0.056 |  |  |  |
| EOC - EOF | 4.677 | 102 | < .001 | 0.258 | 0.056 |  |  |  |
| Age Group (Younger Adults - Older Adults) $\times$ Condition |  |  |  |  |  | 4.80 | .060 | .015 |
| EOF | -1.250 | 91.8 | .215 | -0.127 | 0.101 |  |  |  |
| EOC | -2.607 | 91.8 | .011 | -0.264 | 0.101 |  |  |  |
| ECC | -4.838 | 91.8 | < .001 | -0.490 | 0.101 |  |  |  |
| ECSS | -4.556 | 91.8 | < .001 | -0.462 | 0.101 |  |  |  |

*Note*. The dependent variable was Velocity-RMS. Paired *t*-tests were corrected with Tukey-method to control for familywise errors. Sphericity was corrected using Greenhouse-Geisser method.

EOF = Eyes-Open, Firm Surface; EOC = Eyes-Open, Compliant Surface; ECC = Eyes-Closed, Compliant Surface; ECSS = Eyes-Closed with Sound Suppression.

**S.3 Detailed Scatterplots (Accompanies Figure 5)**

## Correlation Scatterplots

Note – these data were winsorized before being plotted.

*Figure S.3.1.* Scatterplot illustrating the associations between posturography measures (i.e., COP path length, cm) and perceptual thresholds (i.e., m/s^2^ for heave, and deg/s^2^ for pitch) in, A) older adults, and B) younger adults. Points represent individual participant data. Blue lines represent regression lines. Correlations were Bonferroni-corrected. **p* < .05.

*Note.* EOF = Eyes Open, Firm Surface; EOC = Eyes Open, Compliant Surface; ECC = Eyes Closed, Compliant Surface; ECSS = Eyes Closed, with Sound Suppression on a Compliant Surface.

## Fischer’s R to Z transformation

To compare correlations obtained in older adults to those in younger adults, we ran a Fisher’s R to Z transformation (Table S.3.1), and further converted those z-values into *p*-values (Table S.3.2) to infer significance (*p* < .05). These results indicate that all significant correlations obtained by the older adults (Figure 5) differed significantly from younger adults’ correlations – except for the EOF and pitch detection association. Note – these data were winsorized before being analyzed

**Table S.3.1**

*p-values obtained on Fischer’s R to Z transformation, comparing the correlations obtained in the older and younger adults.*

|  | EOF | EOC | ECC | ECSS |
| --- | --- | --- | --- | --- |
| Heave Detection | < .001* | < .001* | 0.013* | 0.009* |
| Heave Discrimination | 0.299 | 0.355 | 0.248 | 0.141 |
| Pitch Detection | 0.08 | 0.033* | 0.022* | 0.001* |
| Pitch Discrimination | 0.047* | 0.086 | 0.004* | 0.001* |

*Note.* * = *p* < 0.05

**Table S.3.2**

*z-values obtained on Fischer’s R to Z transformation, comparing the correlations obtained in the older and younger adults.*

|  | EOF | EOC | ECC | ECSS |
| --- | --- | --- | --- | --- |
| Heave Detection | -3.578* | -3.441* | -2.214* | -2.36* |
| Heave Discrimination | -0.527 | 0.372 | -0.682 | -1.078 |
| Pitch Detection | -1.407 | -1.835* | -2.014* | -3.064* |
| Pitch Discrimination | -1.674* | -1.366 | -2.667* | -3.065* |

*Note.* * = *z >* $\pm$1.640

# S.4 Vestibular Thresholds Correlations

We ran a series of Bonferroni-corrected Pearson correlations to examine the relationship between the four vestibular perceptual threshold measures in older adults (Figure S.4.1A, Figure S.4.2A) and younger adults (Figure S.4.1B, Figure S.4.2B), but found no significant association between any two measures.

*Figure S.4.1.* Scatterplot illustrating the associations between the perceptual thresholds (i.e., m/s^2^ for heave, and deg/s^2^ for pitch) in, A) older adults, and B) younger adults. Points represent individual participant data. Blue lines represent regression lines. Red lines represent regression lines for the autocorrelated data. Correlations were Bonferroni-corrected. **p* < .05.

*Figure S.4.2.* Correlation heatmaps illustrating the associations between the perceptual thresholds (i.e., m/s^2^ for heave, and deg/s^2^ for pitch) in, A) older adults, and B) younger adults. Blue squares represent negative correlations and red squares represent positive correlations. Lighter squares represent weaker correlations and darker squares represent stronger correlations. Correlations were Bonferroni-corrected. **p* < .05.

# S.5 Postural Stability and Vestibular Thresholds Regressions

We ran a single multivariate multiple regression to examine the extent to which the four vestibular psychophysical threshold measures (heave detection, heave discrimination, pitch detection, and pitch discrimination) as well as age (as a continuous variable) might *predict* postural outcomes (COP path length) across all four posturography conditions (EOF, EOC, ECC, ECSS).

$$lm(cbind(EOF, EOC, ECC, ECSS) \sim heave detection + heave discrimination + pitch detection + pitch discrimination + Age$$

Before running the analyses, the vestibular psychophysical threshold data were centered, and normality of the dependent variables was ensured using a Kolmogorov-Smirnov test. Ultimately, the multivariate analyses (Table S.5.1) reveled that postural stability, as a whole, was significantly predicted by heave detection (*F*(4, 26) = 4.937, *p* = 0.004) and pitch detection thresholds (*F*(4, 26)= 7.251, *p* < .001). When then ran univariate tests (Table S.5.2) which showed that heave detection thresholds positively predicted, more specifically, COP path lengths in the EOF (*b* = 134.24, *SE* = 30.07, *t* = 4.46, *p* < .001) and EOC (*b* = 126.33, *SE* = 45.41, *t* = 2.78, *p* = .009) conditions. They also showed that pitch detection thresholds positively predicted COP path lengths in the ECC (*b* = 79.30, *SE* = 15.85, *t* = 5.00, *p* < .001) and ECSS (*b* = 96.91, *SE* = 18.58, *t* = 5.22, *p* < .001) conditions. Importantly, Age (and all other predictors) were not significant predictors of postural stability.

**Table S.5.1**

*Results of the multivariate multiple regression (Pillai’s trace) predicting postural stability from vestibular perceptual thresholds and age.*

|  | *df* | Pillai’s Trace | *F* (df) | *p*-value |
| --- | --- | --- | --- | --- |
| Intercept | 1 | .61 | 10.03 (4, 26) | < .001* |
| Heave Detection | 1 | .43 | 4.94 (4, 26) | .004* |
| Heave Discrimination | 1 | .06 | 0.43 (4, 26) | .789 |
| Pitch Detection | 1 | .53 | 7.25 (4, 26) | <.001* |
| Pitch Discrimination | 1 | .18 | 1.40 (4, 26) | .261 |
| Age | 1 | .10 | 0.71 (4, 26) | .590 |

*Note.* The dependent variables were COP path length for the EOF, EOC, ECC, and ECSS conditions, together. EOF = Eyes-Open, Firm Surface; EOC = Eyes-Open, Compliant Surface; ECC = Eyes-Closed, Compliant Surface; ECSS = Eyes-Closed with Sound Suppression. * = *p* < .05

**Table S.5.2**

*Results of the multiple regressions predicting each of the four postural stability measures (EOF, EOCC, ECC, ECSS) separately, from vestibular perceptual thresholds and age.*

| **Predictor** | $\boldsymbol{\beta}$ | ***SE*** | ***t*** | ***p*** | ***F*** | ***df*** | ***p*** | **adj. *R*^2^** |
| --- | --- | --- | --- | --- | --- | --- | --- | --- |
| DV: EOF |  |  |  |  |  |  |  |  |
| Overall model |  |  |  |  | 8.61 | 5, 29 | < .001* | .53 |
| (Intercept) | 14.99 | 2.78 | 5.38 | < .001* |  |  |  |  |
| Heave Detection | 134.24 | 30.07 | 4.46 | < .001* |  |  |  |  |
| Heave Discrimination | -9.94 | 8.41 | -1.18 | 0.25 |  |  |  |  |
| Pitch Detection | 4.23 | 4.36 | 0.97 | 0.34 |  |  |  |  |
| Pitch Discrimination | -0.32 | 0.46 | -0.69 | 0.50 |  |  |  |  |
| Age | 0.05 | 0.05 | 0.91 | 0.37 |  |  |  |  |
|  |  |  |  |  |  |  |  |  |
| DV: EOC |  |  |  |  |  |  |  |  |
| Overall model |  |  |  |  | 8.59 | 5, 29 | < .001* | .53 |
| (Intercept) | 20.37 | 4.2 | 4.85 | < .001* |  |  |  |  |
| Heave Detection | 126.33 | 45.41 | 2.78 | 0.01* |  |  |  |  |
| Heave Discrimination | -12.22 | 12.71 | -0.96 | 0.34 |  |  |  |  |
| Pitch Detection | 12.59 | 6.59 | 1.91 | 0.07 |  |  |  |  |
| Pitch Discrimination | 0.54 | 0.7 | 0.77 | 0.45 |  |  |  |  |
| Age | 0.12 | 0.08 | 1.58 | 0.12 |  |  |  |  |
|  |  |  |  |  |  |  |  |  |
| DV: ECC |  |  |  |  |  |  |  |  |
| Overall model |  |  |  |  | 9.48 | 5, 29 | < .001* | .56 |
| (Intercept) | 53.84 | 10.12 | 5.32 | < .001* |  |  |  |  |
| Heave Detection | 27.79 | 109.29 | 0.25 | 0.80 |  |  |  |  |
| Heave Discrimination | 5.07 | 30.58 | 0.17 | 0.87 |  |  |  |  |
| Pitch Detection | 79.3 | 15.85 | 5 | < .001* |  |  |  |  |
| Pitch Discrimination | 0.04 | 1.68 | 0.02 | 0.98 |  |  |  |  |
| Age | 0.02 | 0.19 | 0.1 | 0.92 |  |  |  |  |
|  |  |  |  |  |  |  |  |  |
| DV: ECSS |  |  |  |  |  |  |  |  |
| Overall model |  |  |  |  | 13.34 | 5, 29 | < .001* | .64 |
| (Intercept) | 55.47 | 11.86 | 4.68 | < .001* |  |  |  |  |
| Heave Detection | 98.81 | 128.13 | 0.77 | 0.45 |  |  |  |  |
| Heave Discrimination | -8.06 | 35.85 | -0.22 | 0.82 |  |  |  |  |
| Pitch Detection | 96.91 | 18.58 | 5.21 | < .001* |  |  |  |  |
| Pitch Discrimination | 2.48 | 1.97 | 1.26 | 0.22 |  |  |  |  |
| Age | 0.06 | 0.22 | 0.25 | 0.8 |  |  |  |  |

*Note*. DV = Dependent Variable; EOF = Eyes-Open, Firm Surface; EOC = Eyes-Open, Compliant Surface; ECC = Eyes-Closed, Compliant Surface; ECSS = Eyes-Closed with Sound Suppression. * = *p* < .05

**S.6 Detailed Correlations**

**Table S.6.1**

*Pearson correlations between VEMP response and vestibular perceptual thresholds in older adults.*

| Variables | *r* | *p* |
| --- | --- | --- |
| cVEMP |  |  |
| Heave Detection | -.55 | .04 |
| Heave Discrimination | .05 | .87 |
| Pitch Detection | -.25 | .40 |
| Pitch Discrimination | -.06 | .85 |
|  |  |  |
|  |  |  |
| oVEMP |  |  |
| Heave Detection | -.11 | .72 |
| Heave Discrimination | .49 | .08 |
| Pitch Detection | .07 | .82 |
| Pitch Discrimination | .47 | .09 |

*Note*. Presence of bilateral VEMP was coded as “1”, unilateral as “0.5”, and bilateral absence of a VEMP response as “0”. Only 14 out of the 18 older adult participants in this study completed VEMP testing.

**Table S.6.2**

*Pearson correlations between VEMP response and COP path length in older adults*

| Variables | *r* | *p* |
| --- | --- | --- |
| cVEMP |  |  |
| EOF | .18 | .54 |
| EOC | .16 | .59 |
| ECC | -.29 | .32 |
| ECSS | -.17 | .57 |
|  |  |  |
|  |  |  |
| oVEMP |  |  |
| EOF | -.03 | .92 |
| EOC | .13 | .66 |
| ECC | .26 | .38 |
| ECSS | .31 | .28 |

*Note*. Presence of bilateral VEMP was coded as “1”, unilateral as “0.5”, and bilateral absence of a VEMP response as “0”. Only 14 out of the 18 older adult participants in this study completed VEMP testing.

EOF = Eyes-Open, Firm Surface; EOC = Eyes-Open, Compliant Surface; ECC = Eyes-Closed, Compliant Surface; ECSS = Eyes-Closed with Sound Suppression.
